# Poultry Ownership Associated with Increased Risk of Child Diarrhea: Cross-Sectional Evidence from Uganda

**DOI:** 10.4269/ajtmh.19-0012

**Published:** 2020-01-20

**Authors:** Ayse Ercumen, Chris Prottas, Angela Harris, Angelique Dioguardi, Greg Dowd, Raymond Guiteras

**Affiliations:** 1 Forestry and Environmental Resources, North Carolina State University, Raleigh, North Carolina;; 2 The Water Trust, New York, New York;; 3 Civil, Construction and Environmental Engineering, North Carolina State University, Raleigh, North Carolina;; 4 The Water Trust, Kampala, Uganda;; 5 Agricultural and Resource Economics, North Carolina State University, Raleigh, North Carolina

## Abstract

Domestic animals have been associated with enteric infections in young children and can also be carriers of respiratory viruses. We conducted a cross-sectional assessment of health outcomes in children aged < 5 years associated with animal presence among 793 rural households in Uganda. We recorded the 2-week prevalence of diarrhea and respiratory infections in children, and the number of cows, poultry, sheep/goats, and pigs in the household. We used generalized linear models with robust standard errors to estimate the prevalence ratio (PR) for diarrhea and respiratory infections associated with households owning the above- versus below-median number of animals. We conducted unadjusted and adjusted analyses controlling for socioeconomic, water, sanitation, and hygiene indicators. Children in households with the above-median number (> 5) of poultry had 83% higher diarrhea prevalence than those with ≤ 5 poultry (adjusted PR = 1.83 [1.04, 3.23], *P* = 0.04). Children in households with the above-median number (> 2) of cows had 48% lower prevalence of respiratory infection than those with ≤ 2 cows (adjusted PR = 0.52 [0.35, 0.76], *P* < 0.005). There were no other significant associations between domestic animals and child health. Studies should assess if barring chickens from indoor living quarters and sanitary disposal of chicken and other animal feces can reduce childhood zoonotic infections.

## INTRODUCTION

Fecal contamination from animal sources is increasingly recognized as a risk factor for enteric infections among young children in low-income countries, where domestic animals are often kept in close proximity to living quarters.^1^ Molecular microbial source-tracking methods that allow differentiating between contamination of human versus animal origin have revealed widespread presence of animal fecal markers in the domestic environment in low-income countries.^2–4^ A study in India found that animal fecal markers detected in stored drinking water and on caregiver and child hands was associated with an over 4-fold increase in the odds of diarrhea in children aged < 5 years.^5^ Two of the six leading pathogens associated with moderate-to-severe diarrhea in children in the Global Enteric Multicenter Study (*Cryptosporidium* and *Campylobacter*) have animal hosts.^1,6,7^ On the other hand, an analysis of demographic health survey data from 30 countries in sub-Saharan Africa did not find an association between animal ownership and child diarrhea but found slightly increased risk of child mortality associated with animal ownership; the study also revealed significant heterogeneity between countries.^8^

It is possible that different domestic animals (e.g., ruminant versus avian) vary in the level of human health risk they pose as they are hosts to different zoonotic pathogens.^9^ For example, poultry and cattle are carriers of *Campylobacter* and *Salmonella*, but cattle also carry *Escherichia coli* O157:H7 and *Cryptosporidium*.^10–12^ A systematic review and meta-analysis found more clear relationships between animal exposure and enteric infections when a causal pathogen was identified and specific animal–pathogen pairs were investigated; the strongest relationship was between *Campylobacter* and poultry, with an almost 3-fold increase in the odds of *Campylobacter* infection associated with poultry exposure.^13^

Most studies of child exposure to domestic animals to date have focused on enteric infections. Chickens can transmit respiratory infections to humans.^14–16^ In addition, respiratory infections in children have been linked to diarrheal episodes. Malnutrition, which can result from diarrhea, is a risk factor for acute lower respiratory infections, and strains on the body from diarrhea, such as stress on the immune system and loss of micronutrients, can also put children at increased risk of respiratory infection.^17^ Recent diarrheal episodes have been associated with increased risk of pneumonia and acute lower respiratory infections among young children.^17–19^ On the other hand, animal ownership can improve the nutritional status, both through consumption of nutrient-rich animal-based foods or through income generation and consequently increased purchasing power for food items.^20^ Improved nutrition can, in turn, help fight off infections by boosting immune function.^21^ We conducted an analysis of the relationship between ownership of different domestic animals (cows, poultry, and sheep/goats) and diarrhea and respiratory infection in children aged < 5 years among rural households in Uganda.

## MATERIALS AND METHODS

We used data from an existing cross-sectional survey conducted in April 2018 among 1,235 households in 22 villages in Kiryandongo and Masindi districts of Uganda. The participants were selected for the survey based on their anticipated participation in an upcoming water, sanitation, and hygiene program implemented by the Water Trust. The selection criteria included that the communities were rural and had low levels of reliable water access and sanitation. For our analysis, we excluded households with no children aged < 5 years, yielding a sample size of 793 households with a total of 1,336 children aged < 5 years.

Enumerators hired and trained by a third-party monitoring and evaluation agency, Lida Africa, visited participants in their homes to conduct a structured questionnaire and spot-check observations. They recorded the caregiver-reported 2-week prevalence of diarrhea (defined as three loose, watery, or bloody stools in a 24-hour period) and respiratory infections in children aged < 5 years. They also recorded the self-reported number of cows, poultry, sheep/goats, and pigs owned by the household. Enumerators also collected self-reported data on potential confounding factors such as demographic and socioeconomic indicators (the number of people living in the household, whether all school-aged children are attending school, whether the female household head/spouse can read and write, and main fuel type used for cooking), household assets (whether household members own a radio, mobile phone(s), and at least one pair of shoes for every member), and water, sanitation, and hygiene indicators (water source type, functionality and distance, latrine presence and type, and participants’ reported knowledge about key times for handwashing). We augmented the self-reported questionnaire data with spot-check observations on water, sanitation, hygiene, and socioeconomic indicators; spot checks during unannounced visits can provide a rapid and unbiased method to capture day-to-day household practices and conditions.^22^ Enumerators observed the household’s handwashing facility to check for the presence of water and soap, inspected the compound for human and animal feces in the living area for young children, and observed the materials of the walls and roof, and the ventilation status of the kitchen.

To quantify households’ socioeconomic status, we calculated a poverty probability index (PPI^®^) that has been specifically developed and locally validated for the Ugandan setting based on data from the 2012 to 2013 National Household Survey conducted by the Uganda Bureau of Statistics.^23^ The PPI estimates the probability that a household is below the poverty line based on 10 questions on household assets and sociodemographic characteristics, including the number of people living in the household; whether all school-aged children are attending school; whether the female head/spouse can read and write; whether household members own a radio, mobile phone(s), and at least one pair of shoes for every member; the materials of the walls and roof; main fuel type used for cooking; and the type of toilet used by the household. We also calculated the total assets of each household by summing up their reported savings, the reported value any businesses owned by the household, and the estimated value of any owned land and domestic animals. We estimated the value of an acre of land at 2,000,000 Ugandan shillings (USD 527), the value of a cow at 900,000 shillings (USD 237), the value of a pig at 500,000 shillings (USD 132), the value of sheep/goats at 150,000 shillings (USD 40), and the value of a chicken at 30,000 shillings (USD 8), based on the local market prices at the time of the study.

We estimated the prevalence ratio (PR) for diarrhea and respiratory infection in children aged < 5 years associated with households owning the above- versus below-median number of any animal, cows, poultry, and sheep/goats. We selected this exposure definition as it captures a higher exposure representing what is a large number of animals in this specific study population, and it also divides the dataset into optimally sized exposure groups to maximize statistical power to detect between-group differences. In addition, we also estimated the PR associated with increasing number of animals owned (i.e., PR for each additional cow and sheep/goat and for every 10 additional chickens/birds). We did not estimate PRs for pig ownership because of the small number of households owning pigs. We estimated PRs using generalized linear models with a Poisson error distribution with a log link function and robust standard errors accounting for clustering of health outcomes within study villages.^24,25^ We estimated unadjusted PRs as well as adjusted PRs controlling for socioeconomic, water, sanitation, and hygiene indicators. We considered the following potential confounders: village of residence; total value of assets; PPI score (which includes sanitation access); improved water access; water source functionality and distance; handwashing reported before food preparation, after defecation, after handling feces, and after handling animals; and (for respiratory infection) ventilation status of the kitchen. We included all covariates that showed an association with the outcome of interest at the *P* < 0.2 level in final multivariable models.^26^ To further assess potential confounding by socioeconomic status, we investigated the relationship between animal ownership and socioeconomic status by comparing the number of animals owned across PPI quartiles with one-way analysis of variance (ANOVA). To assess exposure to animal feces as an intermediate outcome, we conducted a χ^2^ test to compare the prevalence of observed feces in the living area between households with the above- versus below-median number of animals. A checklist on study elements has been provided as per the Strengthening the Reporting of Observational Studies in Epidemiology (STROBE) guidelines (see Supplemental Text 1).^27^

The sample size for our analysis was determined by the number of households with available survey data and a child aged < 5 years. Post hoc calculations of minimum detectable effect based on our recorded 2-week prevalence of diarrhea and respiratory infections indicated that our sample size of 1,336 children would allow 80% power to detect a 62% relative change in diarrhea prevalence and a 50% relative change in respiratory infection prevalence between children living in households with the above- versus below-median number of animals, with a two-sided α of 0.05 and an intracluster correlation coefficient of 0.005 for children in the same village.^28^

### Ethics.

The data used for this analysis were collected to serve as baseline for a programmatic evaluation by the Water Trust, and the analysis was conducted using de-identified data. The reported analysis was therefore determined to be exempt from ethical review by the human subjects committee of North Carolina State University. Verbal informed consent was obtained before the administration of each survey.

## RESULTS

### Household characteristics.

Approximately 70% of households had access to an improved water source, with approximately 50% of households drawing water from a tubewell or borehole (Table 1). Three quarters of households reported that their main water point was at least partly functional, and half had their main water point less than 0.5 km away. Approximately 80% of households owned a latrine; more than 90% of latrines were uncovered pit latrines. The vast majority (97%) of participants listed “before eating” as a key moment for handwashing, and 51% listed “after defecating,” whereas < 25% of participants listed “before preparing food” as a key handwashing moment, and < 10% listed “after handling child feces” or “after working with animals.” Only 2% of participants had a designated handwashing facility with water and soap observed. Approximately 17% of households had animal or human feces observed in young children’s living area. Cow dung was used as fuel; 92% of households reported using rudimentary materials (firewood, cow dung, or grass/reeds) as their main fuel for cooking.

**Table 1:** Demographic, socioeconomic, and water, sanitation, and hygiene indicators (*N* = 793)

	% (*n*)
Demographics and socioeconomics	
Oldest female head/spouse can read and write with understanding in any language	38.3 (302)
Wall material	
Unburnt bricks with mud, mud and poles, or other	69.1 (548)
Unburnt bricks with cement, wood, tin/iron sheets, concrete/stones, burnt stabilized bricks, or cement blocks	30.9 (245)
Roof material	
Thatch, banana leaves/fibers, grass papyrus, or tin	65.1 (516)
Iron sheets, concrete, tiles, asbestos, or other	34.9 (277)
Main fuel used for cooking	
Firewood, cow dung, or grass (reeds)	92.2 (731)
Charcoal, paraffin stove, gas, biogas, electricity, or other	7.8 (62)
Number of mobile phones owned by household members (at household level)	
None	47.7 (378)
One	40.0 (317)
Two or more	12.4 (98)
Household owns radio	66.7 (529)
Every member of the household has at least one pair of shoes	73.1 (580)
Water, sanitation, and hygiene indicators	
Water source	
Improved	
Tube well/borehole	53.0 (420)
Protected dug well/shallow well	9.1 (72)
Protected spring	5.7 (45)
Public tap/standpipe	3.4 (27)
Rainwater collection	0.9 (7)
Unimproved	
Unprotected source/surface water	28.0 (222)
Main water point functional or partly functional	75.9 (602)
Main water source < 0.5 km away	46.1 (364)
Time to fetch water < 30 minutes roundtrip	36.6 (290)
Household owns a latrine	79.7 (629)
Latrine type	
Uncovered pit latrine without superstructure	35.6 (224)
Uncovered pit latrine with superstructure	55.0 (346)
Covered pit latrine without superstructure	4.8 (30)
Covered pit latrine with superstructure	2.5 (16)
Ventilated improved pit latrine	2.1 (13)
Household has handwashing facility with water and soap	1.9 (12)
Respondent-reported key moments for handwashing include	
Before eating	97.0 (769)
Before preparing food	21.2 (168)
After defecating	51.0 (404)
After handling child feces	7.9 (63)
After handling animals	7.8 (62)
Animal or human feces observed on the ground inside the home	16.9 (134)

### Animal ownership.

Approximately two-thirds of households owned at least one animal, with 17% owning cows, 57% owning chickens/birds, 32% owning sheep/goats, and 3% owning pigs (Table 2). Households with animals owned a median of two cows (interquartile range [IQR]: 1–4, range: 1–60), five chickens/birds (IQR: 3–8, range: 1–100), two sheep/goats (IQR: 1–3, range: 1–33), and two pigs (IQR: 1–3, range: 1–4). Animal ownership was positively associated with socioeconomic status as measured by the PPI; the number of animals owned progressively increased from the bottom socioeconomic quartile in our dataset to the top quartile (*P*-value = 0.03) (Table 3). This was mostly driven by poultry; the bottom socioeconomic quartile owned an average of 2.7 chickens/birds, whereas the top quartile owned an average of 4.8 chickens/birds (*P*-value = 0.01). There was no association between socioeconomic quartile and the number of cows, sheep/goats, or pigs owned (*P*-value> 0.05). The prevalence of observed feces within the living area was 21% among households with an above-median number of animals versus 15% among those with a below-median number of animals (χ^2^ *P*-value = 0.03).

**Table 2:** Animal ownership (*N* = 793)

	Households owning animal, % (*n*)	Number of animals*, median (interquartile range)
Any animal	64.8 (514)	6 (3–10)
Cows	17.3 (137)	2 (1–4)
Poultry	56.6 (449)	5 (3–8)
Sheep/goats	31.8 (252)	2 (1–3)
Pigs	3.3 (26)	2 (1–3)

* Among households that have at least one animal.

**Table 3:** Animal ownership by categories of poverty probability index (PPI)* (*N* = 793)

Mean number of animals (range)	Top quartile	Second quartile	Third quartile	Bottom quartile	ANOVA *P*-value
Any animal	6.8 (0–103)	5.5 (0–48)	5.2 (0–33)	4.3 (0–61)	0.03
Cows	1.0 (0–60)	0.7 (0–15)	0.4 (0–8)	0.7 (0–30)	0.28
Poultry	4.8 (0–100)	3.7 (0–30)	3.6 (0–20)	2.7 (0–20)	0.01
Sheep/goats	0.8 (0–15)	1.0 (0–33)	1.1 (0–11)	0.9 (0–11)	0.54
Pigs	0.1 (0–4)	0.1 (0–4)	0.1 (0–2)	0.0 (0–2)	0.09

* Poverty probability index estimates the probability that a household is below the poverty line based on the number of people living in the household; whether all school-aged children are attending school; whether the female head/spouse can read and write; whether household members own a radio, mobile phone(s), and at least one pair of shoes for every member; the materials of the walls and roof; main fuel type used for cooking; and type of toilet used by the household.

### Diarrhea and respiratory infection in children aged < 5 years.

The caregiver-reported 2-week prevalence of symptoms among children aged < 5 years was 5.2% for diarrhea and 8.5% for respiratory infection. In unadjusted bivariate analyses, children in households with the above-median number of cows had a lower prevalence of diarrhea and respiratory infection, and children in households with the above-median number of poultry had a lower prevalence of respiratory infection (Table 4). In adjusted models controlling for potential confounders, children in households that owned more than the median number (> 5) of poultry had 83% higher diarrhea prevalence than those with ≤ 5 poultry (adjusted PR = 1.83 (1.04, 3.23), *P*-value = 0.04, Table 4). Children in households with more than the median number (> 2) of cows had 48% lower prevalence of respiratory infection than those with ≤ 2 cows (adjusted PR = 0.52 (0.35, 0.76), *P*-value < 0.005, Table 4). The analysis using the number of animals as the independent variable yielded similar effect estimates. In unadjusted analyses, children exposed to increasing number of cows had a lower prevalence of diarrhea and respiratory infection, whereas children exposed to increasing number of poultry had a lower prevalence of respiratory infection (Table 5). In adjusted models, each additional cow was associated with 19% lower prevalence of respiratory infection (adjusted PR = 0.81 (0.66, 0.98), *P*-value = 0.03, Table 5). The number of poultry was not significantly associated with diarrhea, but the direction and magnitude of the effect estimate suggested an increase in diarrhea prevalence associated with every additional 10 chickens/birds (adjusted PR = 1.29 [0.85, 1.95], *P*-value = 0.23, Table 5). There were no other significant associations between animals and health outcomes in adjusted analyses.

**Table 4:** Two-week prevalence of diarrhea and respiratory infection in children aged < 5 years associated with owning the above- vs. below-median number of animals (*N* = 1,336)

	Unadjusted		Adjusted*	
	PR (95% CI)	*P*-value	PR (95% CI)	*P*-value
Diarrhea				
Animals (> 6 vs. ≤ 6)	0.86 (0.45, 1.62)	0.63	1.18 (0.57, 2.44)	0.65
Cows (> 2 vs. ≤ 2)	0.27 (0.08, 0.98)	0.05	0.41 (0.08, 2.09)	0.28
Poultry (> 5 vs. ≤ 5)	1.09 (0.58, 2.05)	0.79	1.83 (1.04, 3.23)	0.04
Sheep/goats (> 2 vs. ≤ 2)	0.48 (0.14, 1.59)	0.23	0.41 (0.13, 1.32)	0.14
Respiratory infection				
Animals (> 6 vs. ≤ 6)	0.37 (0.21, 0.64)	< 0.005	0.56 (0.32, 0.99)	0.05
Cows (> 2 vs. ≤ 2)	0.51 (0.28, 0.92)	0.02	0.52 (0.35, 0.76)	< 0.005
Poultry (> 5 vs. ≤ 5)	0.40 (0.23, 0.69)	< 0.005	0.62 (0.36, 1.07)	0.09
Sheep/goats (> 2 vs. ≤ 2)	0.93 (0.46, 1.84)	0.83	1.31 (0.75, 2.27)	0.34

PR = prevalence ratio; CI = confidence interval.

* We considered the following adjustment covariates: Village of residence; total value of assets; poverty score (which includes sanitation access); improved water access; water source functionality and distance; handwashing reported before food preparation, after defecation, after handling child feces, and after handling animals; and (for respiratory infection) ventilation status of the kitchen. Covariates associated with each outcome at *P* < 0.2 level in bivariate assessment were included in the adjusted models.

**Table 5:** Two-week prevalence of diarrhea and respiratory infection in children aged < 5 years associated with the number of animals owned* (*N* = 1,336)

	Unadjusted		Adjusted†	
	PR (95% CI)	*P*-value	PR (95% CI)	*P*-value
Diarrhea				
Animals	0.73 (0.45, 1.17)	0.19	0.96 (0.64, 1.46)	0.86
Cows	0.75 (0.59, 0.95)	0.02	0.78 (0.58, 1.05)	0.10
Poultry	0.86 (0.54, 1.37)	0.52	1.29 (0.85, 1.95)	0.23
Sheep/goats	0.86 (0.71, 1.04)	0.12	0.89 (0.76, 1.05)	0.17
Respiratory infection				
Animals	0.55 (0.32, 0.93)	0.03	0.70 (0.42, 1.18)	0.18
Cows	0.78 (0.61, 1.00)	0.05	0.81 (0.66, 0.98)	0.03
Poultry	0.47 (0.22, 1.00)	0.05	0.72 (0.38, 1.36)	0.31
Sheep/goats	0.93 (0.79, 1.10)	0.40	1.00 (0.90, 1.12)	0.97

PR = prevalence ratio; CI = confidence interval.

* Prevalence ratio associated with each additional cow and sheep/goat, and every 10 additional animals and chickens.

† We considered the following adjustment covariates: village of residence; total value of assets; poverty score (which includes sanitation access); improved water access; water source functionality and distance; handwashing reported before food preparation, after defecation, after handling child feces, and after handling animals; and (for respiratory infection) ventilation status of the kitchen. Covariates associated with each outcome at *P* < 0.2 level in bivariate assessment were included in the adjusted models.

## DISCUSSION

We found higher risk of diarrhea associated with increasing exposure to poultry in the household compound but not to other animals. Our findings support a growing body of evidence that chicken exposure is a risk factor for childhood enteric infections. The presence of chickens has been linked to increased risk of *Campylobacter* diarrhea in children in Peru.^29^ A molecular analysis of child and chicken feces in Ecuador detected *Campylobacter* spp. in 76% of chicken feces, and *Campylobacter jejuni* genotypes associated with chickens were more frequently isolated from children’s feces than genotypes associated with other domestic animals, implicating chickens as the primary agent of zoonotic *Campylobacter* transmission.^30^

In our study setting, chickens are raised for domestic consumption and local sale of eggs and meat; only a small minority of households have concentrated feeding operations, whereas most households raise free-range local breed chickens (Masindi District Animal Husbandry Officer, personal communication). Although antibiotics and vaccines are used in the concentrated feedlots, most households do not use chemotherapeutic treatment for their chickens (Masindi District Animal Husbandry Officer, personal communication). The Water Trust field staff report that, in our study setting, chickens are not kept in a defined space and are permitted to wander in and out of the house (whereas cows, sheep, goats, and pigs are more likely to be secured, or, if not secured, not permitted to enter the living area), and some families also sleep with their chickens inside their home to reduce the risk of theft. In addition, whereas cows can be relocated to other areas to graze, chickens typically remain in/near the compound and roam the compound area scavenging for food, scattering their feces in the process. As a consequence, it is possible that chicken feces are more prevalent in the compound environment than feces of other domestic animals. In a study in Bangladesh, 90% of households had chickens and 87% had chicken feces observed in the courtyard, whereas 69% of households had cows, but only 30% had cow feces in the courtyard.^31^

This is consistent with observational evidence of frequent child exposure to chicken feces from other studies. Observations in Peru and Zimbabwe have shown that young children touch and directly ingest chicken feces scattered in the compound.^32,33^ Although older children can also be exposed to animal feces while they help with animal husbandry chores, for children aged < 5 years, it is likely that exposure primarily results from exploratory hand and mouth contact with feces and/or soil contaminated with feces. A study in Bangladesh has found the presence of chickens and chicken feces in the environment to be associated with increased *E. coli* contamination of courtyard soil, stored drinking water, and stored food; contamination associated with chickens was more pronounced than contamination associated with cows, goats, and sheep.^31^

Interventions to corral chickens in an attempt to reduce child exposure to chicken feces have not succeeded in reducing infections. In Peru, corralling chickens increased, rather than decreased, *Campylobacter* diarrhea in children compared with letting them free range.^34^ A study in Ethiopia found reduced child height-for-age Z-scores (HAZ) in households that corralled chickens but no associations between HAZ and other corralled animals; the overall chicken ownership, on the other hand, was associated with improved HAZ.^35^ An alternative to corralling chickens could be to provide designated hygienic play spaces for children that are kept free of animal feces. However, a recent study in Zimbabwe that provided plastic play mats for young children (among other water, sanitation, and hygiene interventions) did not reduce child diarrhea or improve growth.^36^

Even though cow dung was used as cooking fuel in study households, leading to potential contamination of caregiver hands, surfaces, and objects, the presence of cows was not associated with increased prevalence of diarrhea. On the other hand, collecting and setting aside cow dung to be used as fuel may reduce children’s exposure to cow feces in the compound environment. Also, the typical practice of sun-drying cow dung before use can inactivate pathogens through desiccation.^10^ The lack of association we observed between cows and other domestic animals (sheep and goats) and diarrhea is consistent with studies in India and Vietnam that found no relationship between cow exposure and child diarrhea, although the latter study also found no relationship between chicken exposure and diarrhea.^37,38^ It has also been suggested that animal contact can lead to protective immunity, counteracting the effect of zoonotic transmission of enteric pathogens.^39^

We found lower risk of respiratory infection in children associated with increasing exposure to cows. It is possible that cow ownership is associated with increased consumption of dairy products and consequently improved nutritional status.^20^ In our study setting, it is estimated that 90% of cows are raised for meat and 10% for dairy; among dairy cows, 90% of the milk is sold and the rest is reserved for domestic consumption (Masindi District Animal Husbandry Officer, personal communication). An analysis of demographic health survey data from sub-Saharan Africa found that 22 of the 30 countries included in the analysis showed a protective effect of animal ownership against child stunting, indicating improved nutrition.^8^ Improved nutrition, in turn, can reduce the risk of respiratory infection.^19^

We found no increased risk of respiratory infections associated with poultry ownership even though birds are carriers of respiratory pathogens.^40^ Chickens have been associated with bird-to-human transmission of avian influenza,^41^ and elevated antibody titers for influenza A viruses have been detected among agricultural workers and veterinarians exposed to chickens.^14–16^ Respiratory infections have strong seasonality with distinct winter peaks in temperate regions.^42^ In the tropics, where average temperatures are higher with less seasonal variation, the seasonality of respiratory viruses is less well defined. However, studies in the tropics have shown increases in respiratory infections associated with seasonal rainfall and humidity patterns.^43–46^ Seasonal trends and associations with rainfall have also been observed for enteric infections.^47,48^ Our study period in April coincided with the very beginning of the rainy season, which started in late April 2018 in our study area. It is possible that there were no major infections circulating during this month-long study window or that our study duration was not sufficiently long to capture trends in infection. Indeed, disease prevalence in our dataset was substantially lower than previously recorded in the study area. A 2017 survey in the same area found a 2-week prevalence of 11% for diarrhea and 25% for respiratory infection among children aged < 5 years.^49^ The difference could be due to seasonal factors; the 2017 survey was conducted in December just at the beginning of the dry season and may therefore have a higher prevalence of illness from the wet season just ending then, whereas the current survey was conducted at the beginning of the rainy season and may reflect the lower infection prevalence of the dry season. A study conducted during a time of high-intensity transmission or over a long enough period to capture peaks in infection might be better poised to assess associations between animal exposure and infections.

Another limitation of our study is that we relied on caregiver-reported recall of diarrhea and respiratory symptoms over a 2-week period. Whereas reported symptoms could be inaccurate without a clinical diagnosis, self-reported health outcomes are commonly used in epidemiologic studies when clinically confirming infections is not feasible. In addition, although a 2-week recall could have inaccuracies compared with the commonly used shorter recall windows such as 1 week or 2 days,^50,51^ we expect any such inaccuracies to be non-differential with respect to animal ownership (i.e., we do not anticipate that animal ownership will affect the accuracy with which respondents report health endpoints). We therefore assume that any such non-differential misclassification of outcomes would bias our findings toward, rather than away, from the null.^52^ Future studies using shorter recall windows or using clinical specimens to ascertain infections may show more pronounced illness risk associated with chicken exposure. Reported diarrhea also does not differentiate between infections of bacterial, viral, or protozoan etiology. Since different domestic animals are carriers of different pathogens, clinically confirmed infections allowing investigation of pathogen-specific infections and specific animal–pathogen pairs would be expected to reveal clearer associations between animal ownership and health endpoints.^13^

In addition, reported diarrhea symptoms fail to take into account subclinical infections and asymptomatic pathogen carriage. A growing body of literature suggests widespread asymptomatic gut colonization with enteric pathogens among young children in low-income countries.^53^ A study in Bangladesh analyzed stool specimens from children aged < 1 year with versus without symptomatic diarrhea using molecular methods and found that children with no diarrhea had three different pathogens detected in their stool on average, compared with five pathogens among children with diarrhea.^54^ Of the 29 pathogens the study investigated, only seven had a significantly higher prevalence in diarrheal versus non-diarrheal stool samples.^54^ Similarly, a study in Tanzania analyzed stool samples for 19 enteropathogens with molecular methods and found no difference between the number of pathogens detected and the prevalence of any given pathogen between stool samples from children with versus without diarrhea.^55^ The health implications of asymptomatic colonization and subclinical infections are not well understood. Chronic pathogen exposure can result in environmental enteric dysfunction, which is thought to contribute to growth faltering in children.^56,57^ Exposure to domestic animals was associated with markers of environmental enteric dysfunction in children in rural Bangladesh.^58^ Health endpoints that capture asymptomatic pathogen carriage and subclinical infections may allow more nuanced understanding of the health impact of domestic animal exposure among young children; future studies should collect and analyze clinical specimens to detect and quantify pathogen carriage.

Our analysis was observational and is susceptible to confounding, for example, by socioeconomic status, which is typically associated with animal ownership as well as disease prevalence. However, richer households in our dataset owned a larger number of birds such that any confounding from unmeasured socioeconomic factors would likely attenuate and not exaggerate the relationship we observed between poultry ownership and diarrhea. It is also possible that the lower prevalence of respiratory infection associated with increasing number of cows is due to residual confounding from unmeasured socioeconomic factors. However, we used a validated poverty index based on a comprehensive set of indicators to quantify and control for socioeconomic status in our models. Indeed, in unadjusted bivariate models, increasing exposure to cows was associated with a lower prevalence of diarrhea and respiratory infection, whereas increasing exposure to poultry was associated with a lower prevalence of respiratory infection. However, after adjusting for potential confounders including household assets and poverty index, the only negative association that remained significant was the one between cows and respiratory infection, suggesting that this could be a true protective effect. In addition, there was no association between poverty quartile and the number of cows owned.

Finally, our sample size was limited by the number of households with available data, and our analysis was therefore powered for relatively large minimum detectable effects (62% relative change in diarrhea prevalence and 50% relative change in respiratory infection prevalence between children in households with the above- versus below-median number of animals).

Our results indicate increased risk of diarrhea associated with poultry ownership. Although we expect our findings to be generalizable to other settings with similar animal husbandry practices, previous studies indicate substantial heterogeneity on the association between animal exposure and child health. Chickens can provide nutrient-dense foods and have been associated with improved growth in children.^35^ Therefore, it is important to identify strategies to reduce child exposure to chickens and their feces to mitigate the risk of infection while maintaining the nutritional benefits of poultry ownership. It has been suggested that failure to address animal feces can explain why sanitation interventions focused solely on isolating human feces have failed to significantly reduce child exposure to fecal contamination in studies to date.^59,60^ Potential strategies to reduce child exposure to chicken feces could include not keeping chickens in the indoor living quarters and removal and sanitary disposal of chicken feces; studies should assess if these approaches reduce zoonotic infections among young children.
